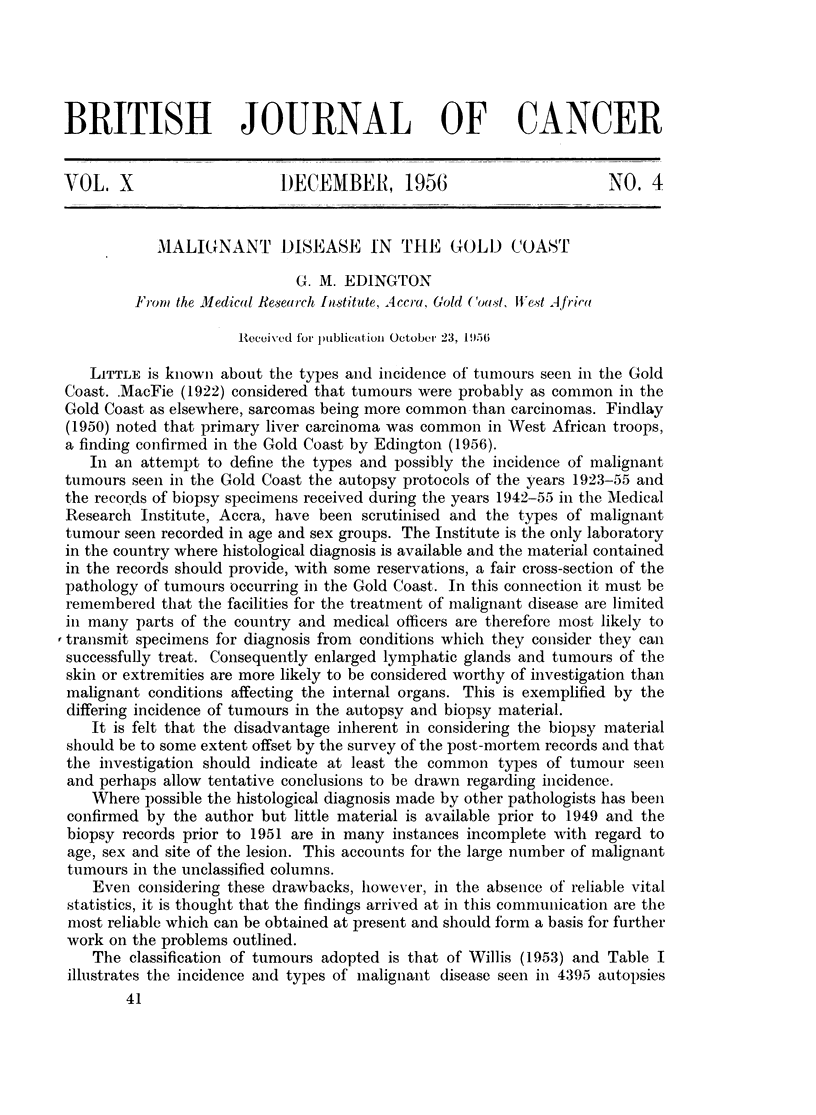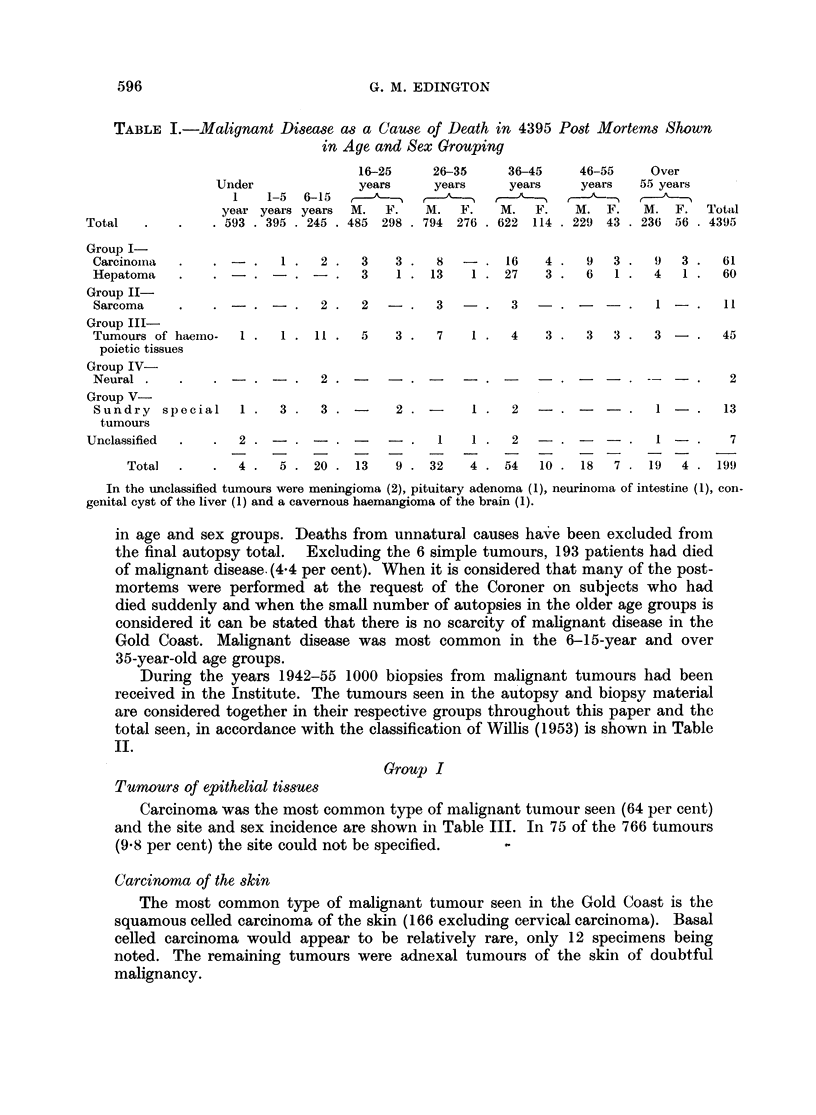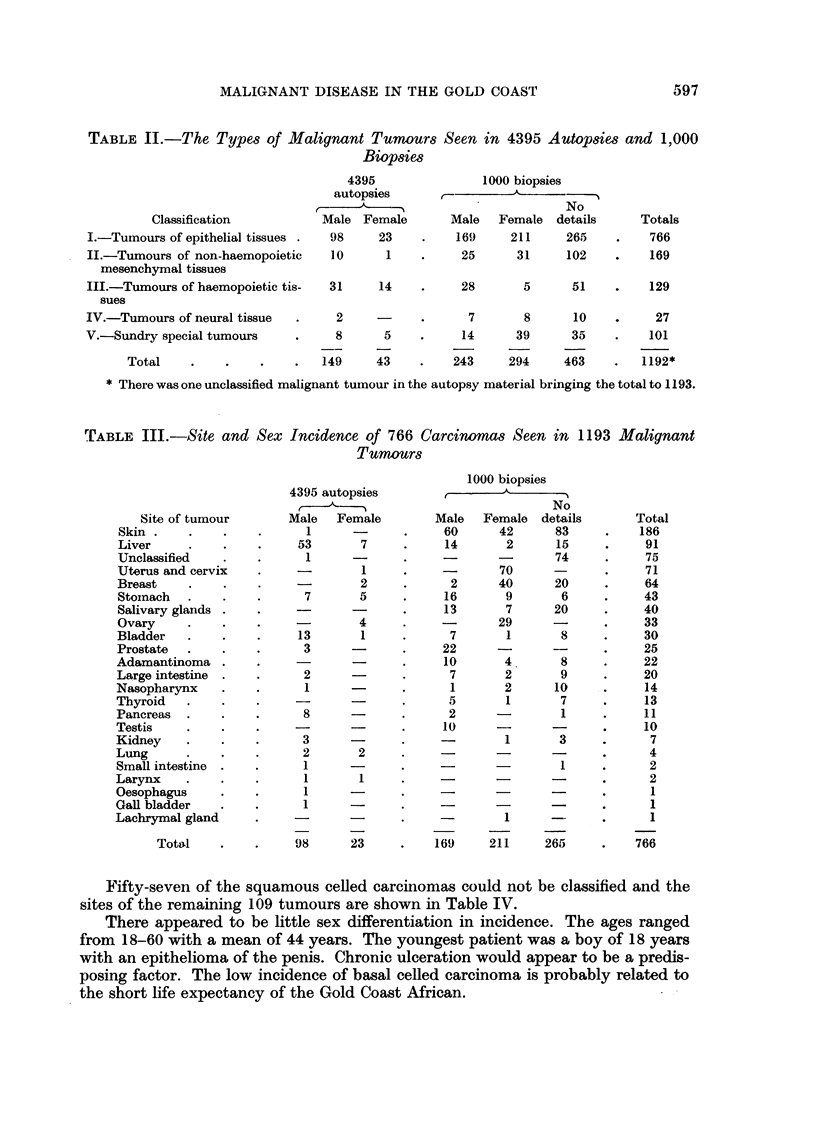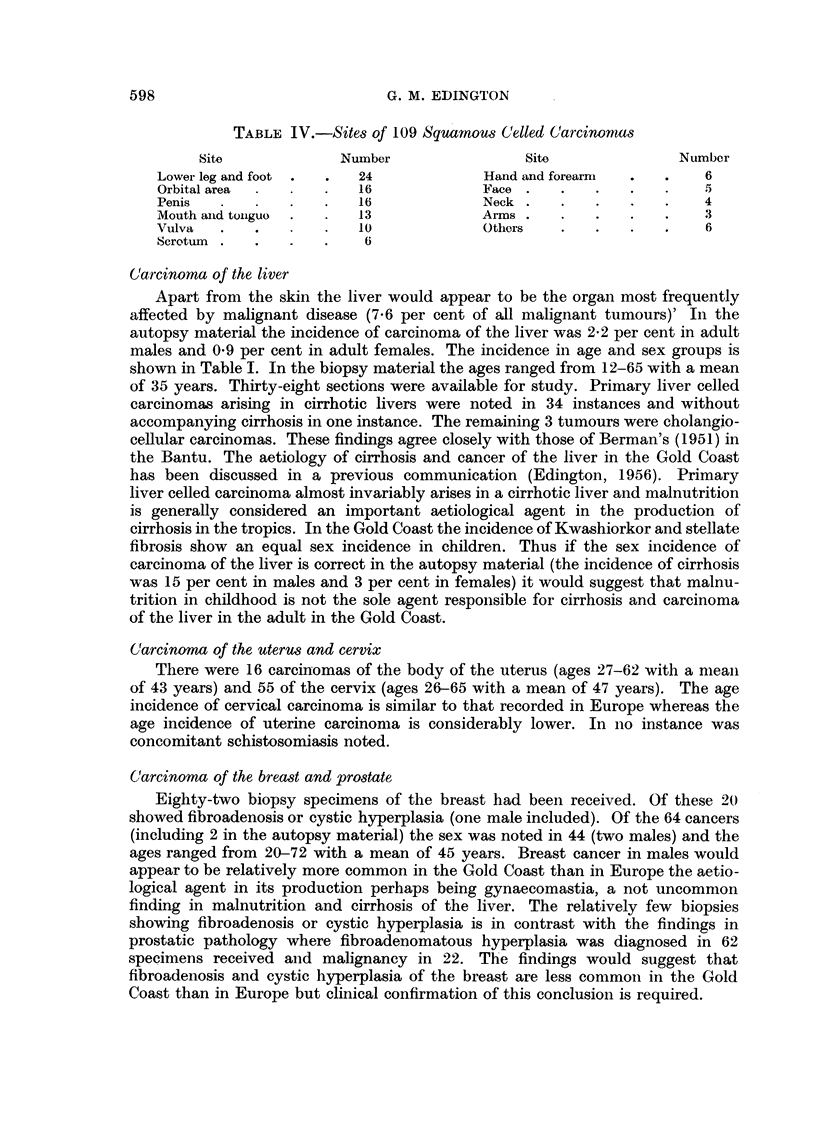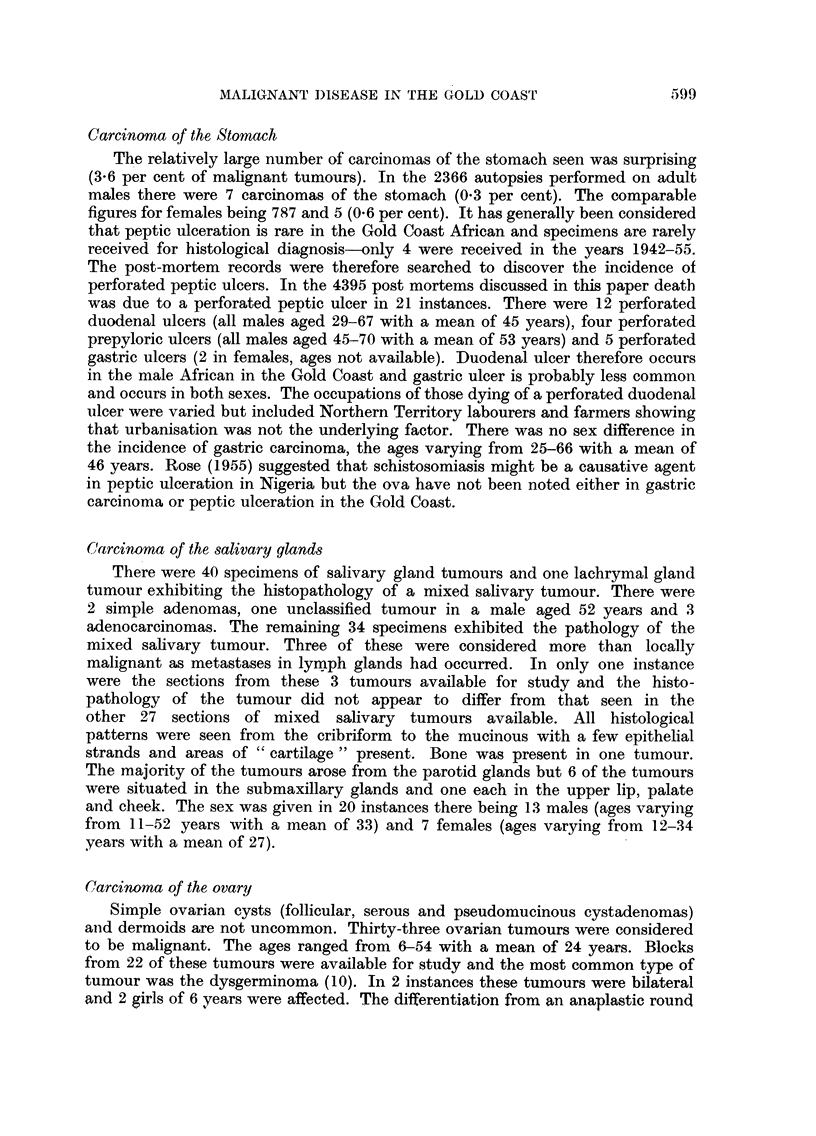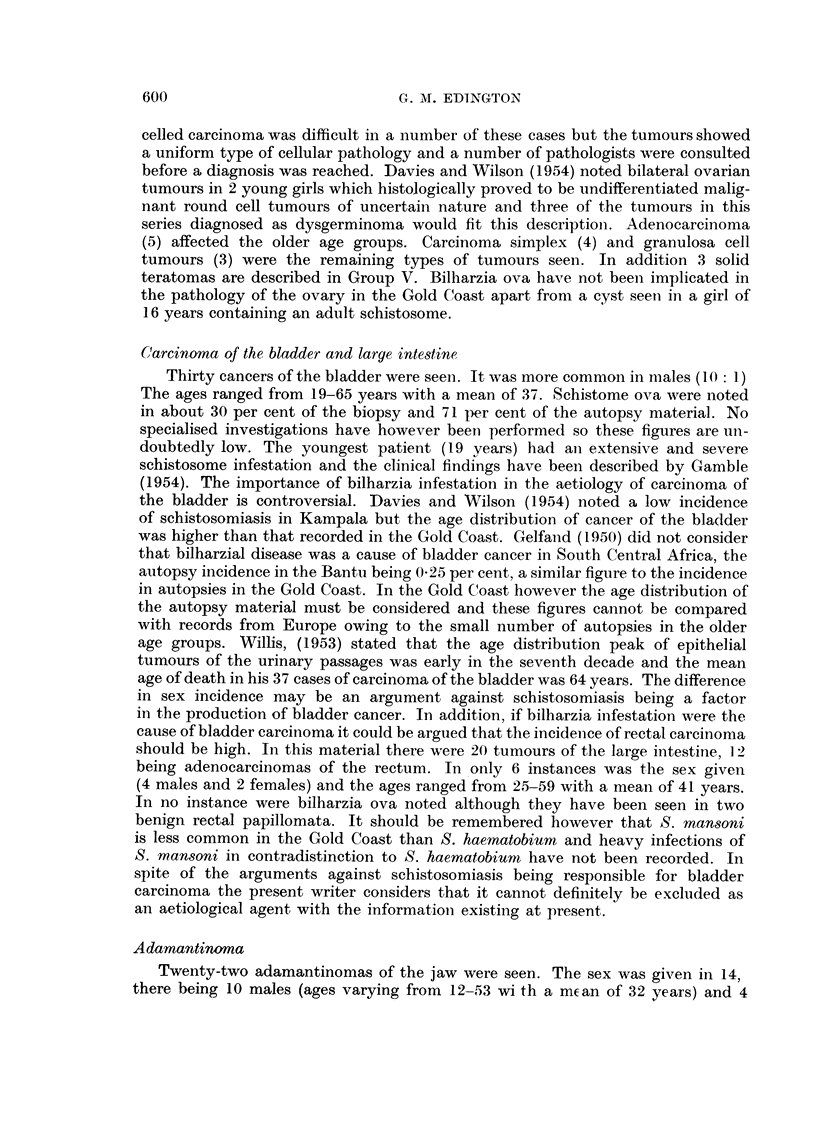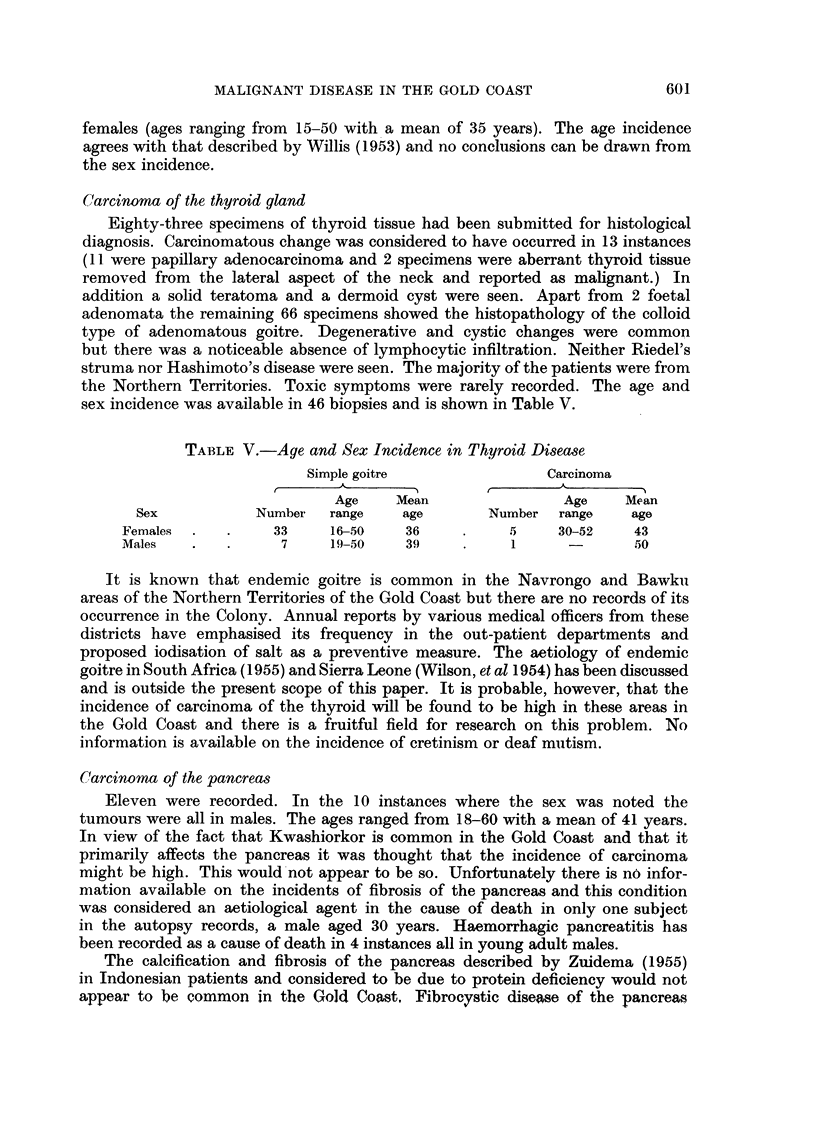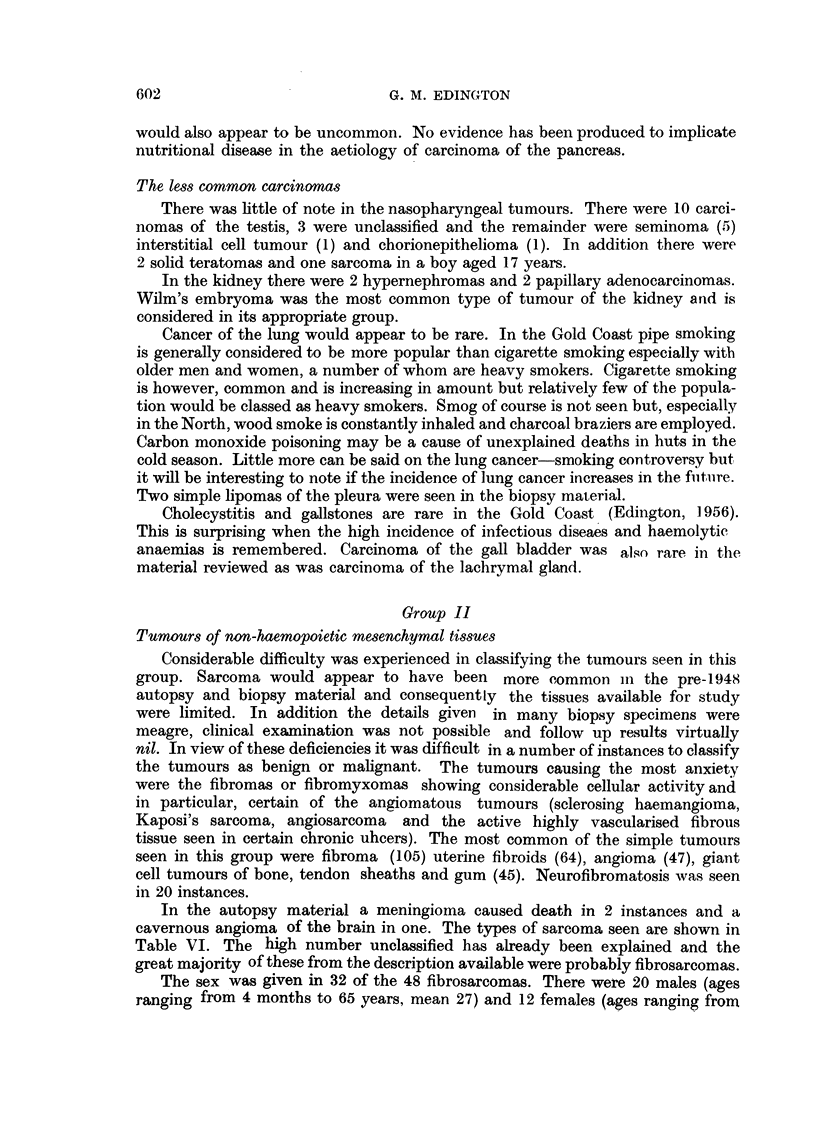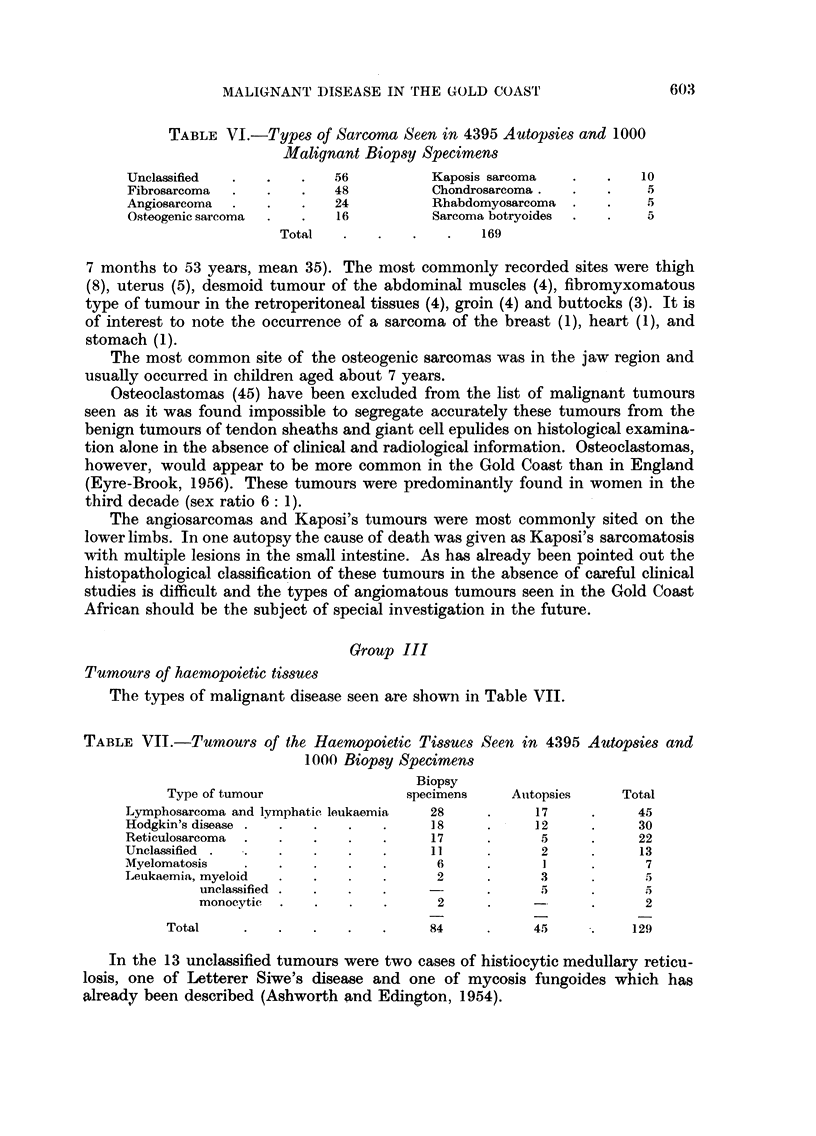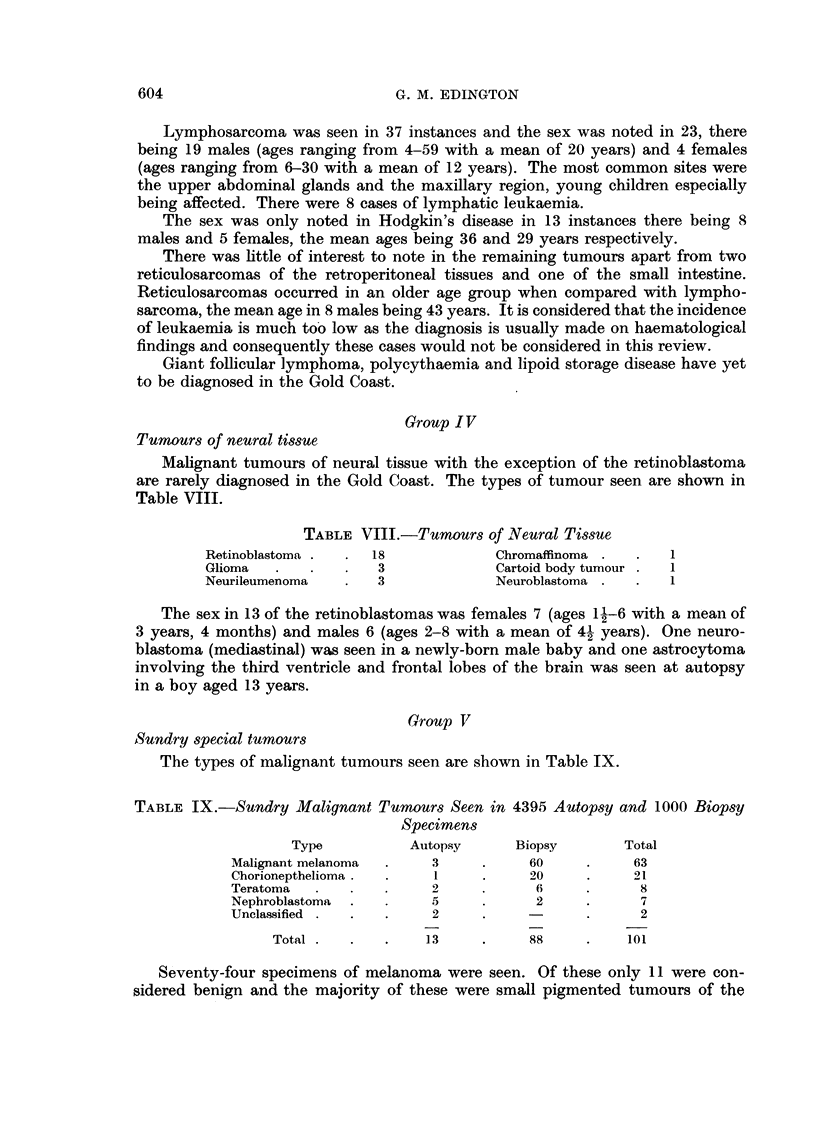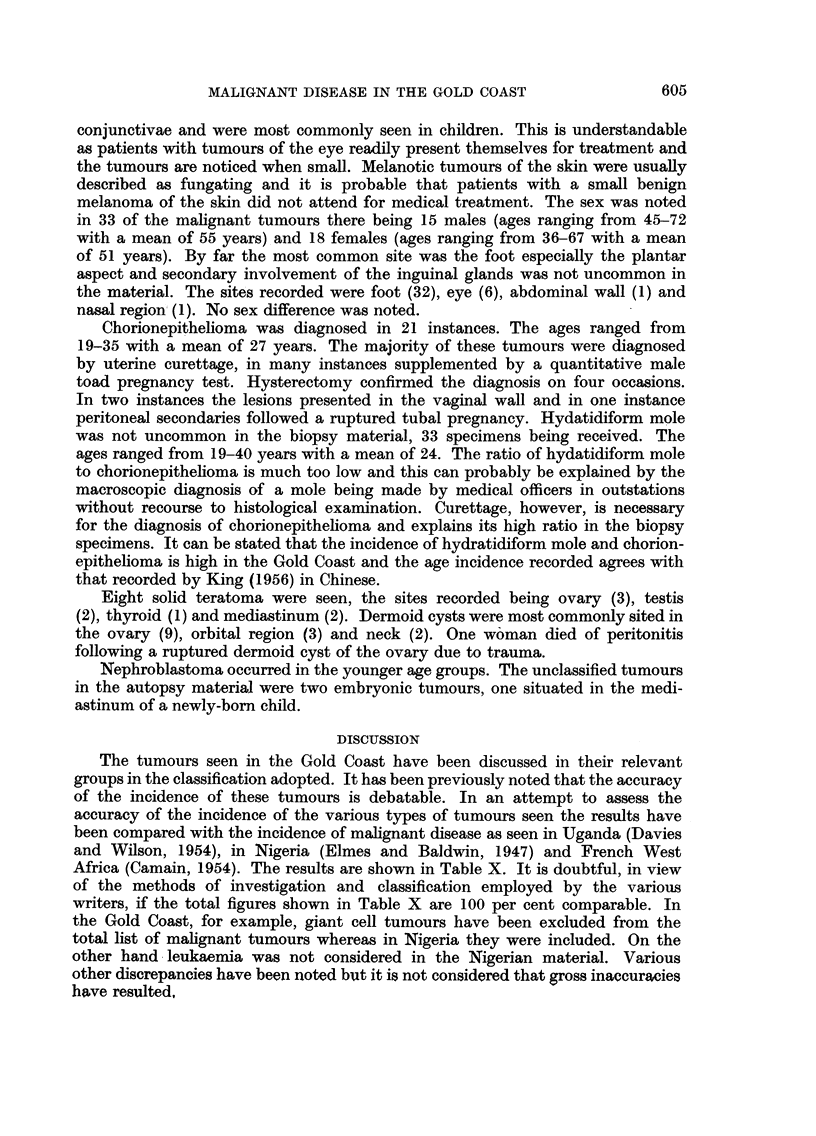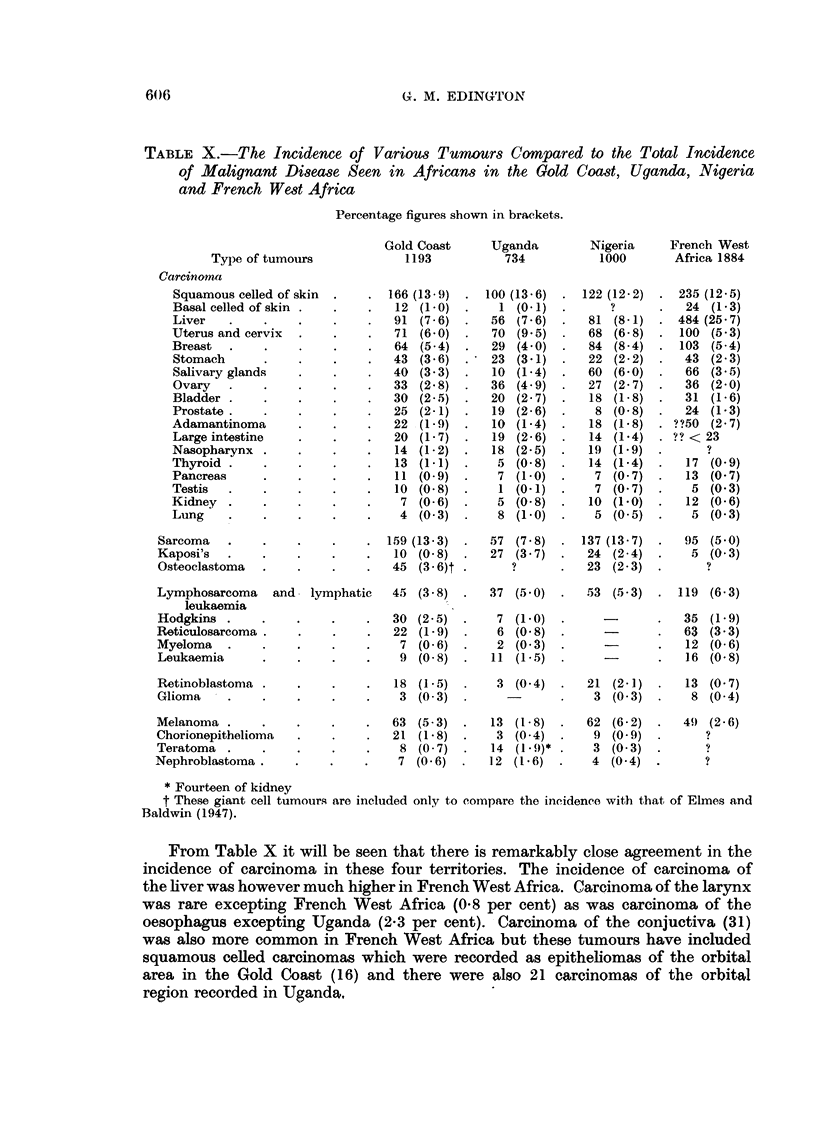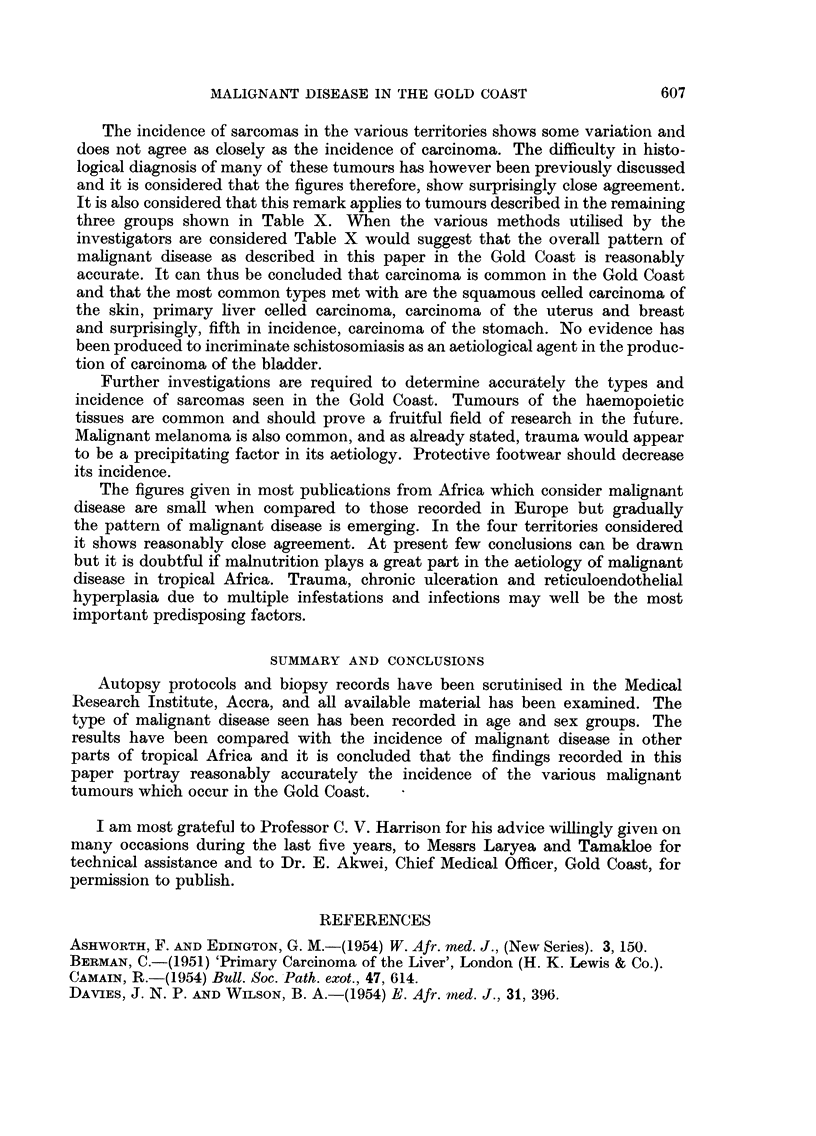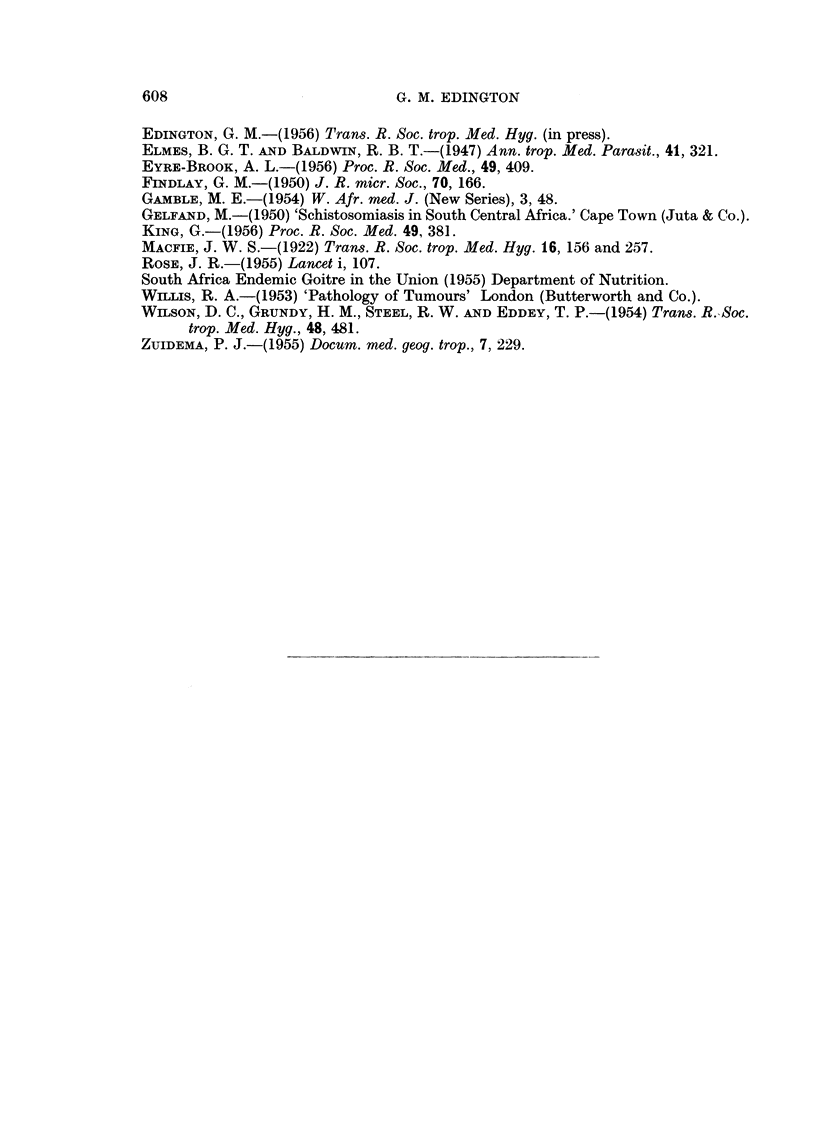# Malignant Disease in the Gold Coast

**DOI:** 10.1038/bjc.1956.72

**Published:** 1956-12

**Authors:** G. M. Edington


### BRITISH JOURNAL OF CANCER

VOL. X DECEMBER, 1956 NO. 4

### MALIGNANT DISEASE IN THE GOLD COAST

G. M. EDINGTON

*From the Medical Research Institute, Accra, Gold Coast, West Africa*

Received for publication October 23, 1956

LITTLE is known about the types and incidence of tumours seen in the Gold Coast. MacFie (1922) considered that tumours were probably as common in the Gold Coast as elsewhere, sarcomas being more common than carcinomas. Findlay (1950) noted that primary liver carcinoma was common in West African troops, a finding confirmed in the Gold Coast by Edington (1956).

In an attempt to define the types and possibly the incidence of malignant tumours seen in the Gold Coast the autopsy protocols of the years 1923–55 and the records of biopsy specimens received during the years 1942–55 in the Medical Research Institute, Accra, have been scrutinised and the types of malignant tumour seen recorded in age and sex groups. The Institute is the only laboratory in the country where histological diagnosis is available and the material contained in the records should provide, with some reservations, a fair cross-section of the pathology of tumours occurring in the Gold Coast. In this connection it must be remembered that the facilities for the treatment of malignant disease are limited in many parts of the country and medical officers are therefore most likely to transmit specimens for diagnosis from conditions which they consider they can successfully treat. Consequently enlarged lymphatic glands and tumours of the skin or extremities are more likely to be considered worthy of investigation than malignant conditions affecting the internal organs. This is exemplified by the differing incidence of tumours in the autopsy and biopsy material.

It is felt that the disadvantage inherent in considering the biopsy material should be to some extent offset by the survey of the post-mortem records and that the investigation should indicate at least the common types of tumour seen and perhaps allow tentative conclusions to be drawn regarding incidence.

Where possible the histological diagnosis made by other pathologists has been confirmed by the author but little material is available prior to 1949 and the biopsy records prior to 1951 are in many instances incomplete with regard to age, sex and site of the lesion. This accounts for the large number of malignant tumours in the unclassified columns.

Even considering these drawbacks, however, in the absence of reliable vital statistics, it is thought that the findings arrived at in this communication are the most reliable which can be obtained at present and should form a basis for further work on the problems outlined.

The classification of tumours adopted is that of Willis (1953) and Table I illustrates the incidence and types of malignant disease seen in 4395 autopsies

TABLE I.—*Malignant Disease as a Cause of Death in 4395 Post Mortems Shown in Age and Sex Grouping*

| | Under 1 year | 1–5 years | 6–15 years | 16–25 years | | 26–35 years | | 36–45 years | | 46–55 years | | Over 55 years | | Total |
|---|---|---|---|---|---|---|---|---|---|---|---|---|---|---|
| | | | | M. | F. | M. | F. | M. | F. | M. | F. | M. | F. | |
| Total | 593 | 395 | 245 | 485 | 298 | 794 | 276 | 622 | 114 | 229 | 43 | 236 | 56 | 4395 |
| Group I— | | | | | | | | | | | | | | |
| Carcinoma | — | 1 | 2 | 3 | 3 | 8 | — | 16 | 4 | 9 | 3 | 9 | 3 | 61 |
| Hepatoma | — | — | — | 3 | 1 | 13 | 1 | 27 | 3 | 6 | 1 | 4 | 1 | 60 |
| Group II— | | | | | | | | | | | | | | |
| Sarcoma | — | — | 2 | 2 | — | 3 | — | 3 | — | — | — | 1 | — | 11 |
| Group III— | | | | | | | | | | | | | | |
| Tumours of haemopoietic tissues | 1 | 1 | 11 | 5 | 3 | 7 | 1 | 4 | 3 | 3 | 3 | 3 | — | 45 |
| Group IV— | | | | | | | | | | | | | | |
| Neural | — | — | 2 | — | — | — | — | — | — | — | — | — | — | 2 |
| Group V— | | | | | | | | | | | | | | |
| Sundry special tumours | 1 | 3 | 3 | — | 2 | — | 1 | 2 | — | — | — | 1 | — | 13 |
| Unclassified | 2 | — | — | — | — | 1 | 1 | 2 | — | — | — | 1 | — | 7 |
| Total | 4 | 5 | 20 | 13 | 9 | 32 | 4 | 54 | 10 | 18 | 7 | 19 | 4 | 199 |

In the unclassified tumours were meningioma (2), pituitary adenoma (1), neurinoma of intestine (1), congenital cyst of the liver (1) and a cavernous haemangioma of the brain (1).

in age and sex groups. Deaths from unnatural causes have been excluded from the final autopsy total. Excluding the 6 simple tumours, 193 patients had died of malignant disease (4·4 per cent). When it is considered that many of the post-mortems were performed at the request of the Coroner on subjects who had died suddenly and when the small number of autopsies in the older age groups is considered it can be stated that there is no scarcity of malignant disease in the Gold Coast. Malignant disease was most common in the 6–15-year and over 35-year-old age groups.

During the years 1942–55 1000 biopsies from malignant tumours had been received in the Institute. The tumours seen in the autopsy and biopsy material are considered together in their respective groups throughout this paper and the total seen, in accordance with the classification of Willis (1953) is shown in Table II.

*Group I*

*Tumours of epithelial tissues*

Carcinoma was the most common type of malignant tumour seen (64 per cent) and the site and sex incidence are shown in Table III. In 75 of the 766 tumours (9·8 per cent) the site could not be specified.

*Carcinoma of the skin*

The most common type of malignant tumour seen in the Gold Coast is the squamous celled carcinoma of the skin (166 excluding cervical carcinoma). Basal celled carcinoma would appear to be relatively rare, only 12 specimens being noted. The remaining tumours were adnexal tumours of the skin of doubtful malignancy.

TABLE II.—*The Types of Malignant Tumours Seen in* 4395 *Autopsies and* 1,000 *Biopsies*

| Classification | 4395 autopsies Male | 4395 autopsies Female | 1000 biopsies Male | 1000 biopsies Female | 1000 biopsies No details | Totals |
|---|---|---|---|---|---|---|
| I.—Tumours of epithelial tissues | 98 | 23 | 169 | 211 | 265 | 766 |
| II.—Tumours of non-haemopoietic mesenchymal tissues | 10 | 1 | 25 | 31 | 102 | 169 |
| III.—Tumours of haemopoietic tissues | 31 | 14 | 28 | 5 | 51 | 129 |
| IV.—Tumours of neural tissue | 2 | — | 7 | 8 | 10 | 27 |
| V.—Sundry special tumours | 8 | 5 | 14 | 39 | 35 | 101 |
| Total | 149 | 43 | 243 | 294 | 463 | 1192* |

* There was one unclassified malignant tumour in the autopsy material bringing the total to 1193.

TABLE III.—*Site and Sex Incidence of* 766 *Carcinomas Seen in* 1193 *Malignant Tumours*

| Site of tumour | 4395 autopsies Male | 4395 autopsies Female | 1000 biopsies Male | 1000 biopsies Female | 1000 biopsies No details | Total |
|---|---|---|---|---|---|---|
| Skin | 1 | — | 60 | 42 | 83 | 186 |
| Liver | 53 | 7 | 14 | 2 | 15 | 91 |
| Unclassified | 1 | — | — | — | 74 | 75 |
| Uterus and cervix | — | 1 | — | 70 | — | 71 |
| Breast | — | 2 | 2 | 40 | 20 | 64 |
| Stomach | 7 | 5 | 16 | 9 | 6 | 43 |
| Salivary glands | — | — | 13 | 7 | 20 | 40 |
| Ovary | — | 4 | — | 29 | — | 33 |
| Bladder | 13 | 1 | 7 | 1 | 8 | 30 |
| Prostate | 3 | — | 22 | — | — | 25 |
| Adamantinoma | — | — | 10 | 4 | 8 | 22 |
| Large intestine | 2 | — | 7 | 2 | 9 | 20 |
| Nasopharynx | 1 | — | 1 | 2 | 10 | 14 |
| Thyroid | — | — | 5 | 1 | 7 | 13 |
| Pancreas | 8 | — | 2 | — | 1 | 11 |
| Testis | — | — | 10 | — | — | 10 |
| Kidney | 3 | — | — | 1 | 3 | 7 |
| Lung | 2 | 2 | — | — | — | 4 |
| Small intestine | 1 | — | — | — | 1 | 2 |
| Larynx | 1 | 1 | — | — | — | 2 |
| Oesophagus | 1 | — | — | — | — | 1 |
| Gall bladder | 1 | — | — | — | — | 1 |
| Lachrymal gland | — | — | — | 1 | — | 1 |
| Total | 98 | 23 | 169 | 211 | 265 | 766 |

Fifty-seven of the squamous celled carcinomas could not be classified and the sites of the remaining 109 tumours are shown in Table IV.

There appeared to be little sex differentiation in incidence. The ages ranged from 18–60 with a mean of 44 years. The youngest patient was a boy of 18 years with an epithelioma of the penis. Chronic ulceration would appear to be a predisposing factor. The low incidence of basal celled carcinoma is probably related to the short life expectancy of the Gold Coast African.

TABLE IV.—*Sites of* 109 *Squamous Celled Carcinomas*

| Site | Number | Site | Number |
|---|---|---|---|
| Lower leg and foot . . | 24 | Hand and forearm . . | 6 |
| Orbital area . . . | 16 | Face . . . . . . | 5 |
| Penis . . . . | 16 | Neck . . . . . . | 4 |
| Mouth and tongue . . | 13 | Arms . . . . . . | 3 |
| Vulva . . . . | 10 | Others . . . . . | 6 |
| Scrotum . . . . | 6 | | |

*Carcinoma of the liver*

Apart from the skin the liver would appear to be the organ most frequently affected by malignant disease (7·6 per cent of all malignant tumours)' In the autopsy material the incidence of carcinoma of the liver was 2·2 per cent in adult males and 0·9 per cent in adult females. The incidence in age and sex groups is shown in Table I. In the biopsy material the ages ranged from 12–65 with a mean of 35 years. Thirty-eight sections were available for study. Primary liver celled carcinomas arising in cirrhotic livers were noted in 34 instances and without accompanying cirrhosis in one instance. The remaining 3 tumours were cholangiocellular carcinomas. These findings agree closely with those of Berman's (1951) in the Bantu. The aetiology of cirrhosis and cancer of the liver in the Gold Coast has been discussed in a previous communication (Edington, 1956). Primary liver celled carcinoma almost invariably arises in a cirrhotic liver and malnutrition is generally considered an important aetiological agent in the production of cirrhosis in the tropics. In the Gold Coast the incidence of Kwashiorkor and stellate fibrosis show an equal sex incidence in children. Thus if the sex incidence of carcinoma of the liver is correct in the autopsy material (the incidence of cirrhosis was 15 per cent in males and 3 per cent in females) it would suggest that malnutrition in childhood is not the sole agent responsible for cirrhosis and carcinoma of the liver in the adult in the Gold Coast.

*Carcinoma of the uterus and cervix*

There were 16 carcinomas of the body of the uterus (ages 27–62 with a mean of 43 years) and 55 of the cervix (ages 26–65 with a mean of 47 years). The age incidence of cervical carcinoma is similar to that recorded in Europe whereas the age incidence of uterine carcinoma is considerably lower. In no instance was concomitant schistosomiasis noted.

*Carcinoma of the breast and prostate*

Eighty-two biopsy specimens of the breast had been received. Of these 20 showed fibroadenosis or cystic hyperplasia (one male included). Of the 64 cancers (including 2 in the autopsy material) the sex was noted in 44 (two males) and the ages ranged from 20–72 with a mean of 45 years. Breast cancer in males would appear to be relatively more common in the Gold Coast than in Europe the aetiological agent in its production perhaps being gynaecomastia, a not uncommon finding in malnutrition and cirrhosis of the liver. The relatively few biopsies showing fibroadenosis or cystic hyperplasia is in contrast with the findings in prostatic pathology where fibroadenomatous hyperplasia was diagnosed in 62 specimens received and malignancy in 22. The findings would suggest that fibroadenosis and cystic hyperplasia of the breast are less common in the Gold Coast than in Europe but clinical confirmation of this conclusion is required.

*Carcinoma of the Stomach*

The relatively large number of carcinomas of the stomach seen was surprising (3·6 per cent of malignant tumours). In the 2366 autopsies performed on adult males there were 7 carcinomas of the stomach (0·3 per cent). The comparable figures for females being 787 and 5 (0·6 per cent). It has generally been considered that peptic ulceration is rare in the Gold Coast African and specimens are rarely received for histological diagnosis—only 4 were received in the years 1942–55. The post-mortem records were therefore searched to discover the incidence of perforated peptic ulcers. In the 4395 post mortems discussed in this paper death was due to a perforated peptic ulcer in 21 instances. There were 12 perforated duodenal ulcers (all males aged 29–67 with a mean of 45 years), four perforated prepyloric ulcers (all males aged 45–70 with a mean of 53 years) and 5 perforated gastric ulcers (2 in females, ages not available). Duodenal ulcer therefore occurs in the male African in the Gold Coast and gastric ulcer is probably less common and occurs in both sexes. The occupations of those dying of a perforated duodenal ulcer were varied but included Northern Territory labourers and farmers showing that urbanisation was not the underlying factor. There was no sex difference in the incidence of gastric carcinoma, the ages varying from 25–66 with a mean of 46 years. Rose (1955) suggested that schistosomiasis might be a causative agent in peptic ulceration in Nigeria but the ova have not been noted either in gastric carcinoma or peptic ulceration in the Gold Coast.

*Carcinoma of the salivary glands*

There were 40 specimens of salivary gland tumours and one lachrymal gland tumour exhibiting the histopathology of a mixed salivary tumour. There were 2 simple adenomas, one unclassified tumour in a male aged 52 years and 3 adenocarcinomas. The remaining 34 specimens exhibited the pathology of the mixed salivary tumour. Three of these were considered more than locally malignant as metastases in lymph glands had occurred. In only one instance were the sections from these 3 tumours available for study and the histopathology of the tumour did not appear to differ from that seen in the other 27 sections of mixed salivary tumours available. All histological patterns were seen from the cribriform to the mucinous with a few epithelial strands and areas of " cartilage " present. Bone was present in one tumour. The majority of the tumours arose from the parotid glands but 6 of the tumours were situated in the submaxillary glands and one each in the upper lip, palate and cheek. The sex was given in 20 instances there being 13 males (ages varying from 11–52 years with a mean of 33) and 7 females (ages varying from 12–34 years with a mean of 27).

*Carcinoma of the ovary*

Simple ovarian cysts (follicular, serous and pseudomucinous cystadenomas) and dermoids are not uncommon. Thirty-three ovarian tumours were considered to be malignant. The ages ranged from 6–54 with a mean of 24 years. Blocks from 22 of these tumours were available for study and the most common type of tumour was the dysgerminoma (10). In 2 instances these tumours were bilateral and 2 girls of 6 years were affected. The differentiation from an anaplastic round celled carcinoma was difficult in a number of these cases but the tumours showed a uniform type of cellular pathology and a number of pathologists were consulted before a diagnosis was reached. Davies and Wilson (1954) noted bilateral ovarian tumours in 2 young girls which histologically proved to be undifferentiated malignant round cell tumours of uncertain nature and three of the tumours in this series diagnosed as dysgerminoma would fit this description. Adenocarcinoma (5) affected the older age groups. Carcinoma simplex (4) and granulosa cell tumours (3) were the remaining types of tumours seen. In addition 3 solid teratomas are described in Group V. Bilharzia ova have not been implicated in the pathology of the ovary in the Gold Coast apart from a cyst seen in a girl of 16 years containing an adult schistosome.

*Carcinoma of the bladder and large intestine*

Thirty cancers of the bladder were seen. It was more common in males (10 : 1) The ages ranged from 19–65 years with a mean of 37. Schistome ova were noted in about 30 per cent of the biopsy and 71 per cent of the autopsy material. No specialised investigations have however been performed so these figures are undoubtedly low. The youngest patient (19 years) had an extensive and severe schistosome infestation and the clinical findings have been described by Gamble (1954). The importance of bilharzia infestation in the aetiology of carcinoma of the bladder is controversial. Davies and Wilson (1954) noted a low incidence of schistosomiasis in Kampala but the age distribution of cancer of the bladder was higher than that recorded in the Gold Coast. Gelfand (1950) did not consider that bilharzial disease was a cause of bladder cancer in South Central Africa, the autopsy incidence in the Bantu being 0·25 per cent, a similar figure to the incidence in autopsies in the Gold Coast. In the Gold Coast however the age distribution of the autopsy material must be considered and these figures cannot be compared with records from Europe owing to the small number of autopsies in the older age groups. Willis, (1953) stated that the age distribution peak of epithelial tumours of the urinary passages was early in the seventh decade and the mean age of death in his 37 cases of carcinoma of the bladder was 64 years. The difference in sex incidence may be an argument against schistosomiasis being a factor in the production of bladder cancer. In addition, if bilharzia infestation were the cause of bladder carcinoma it could be argued that the incidence of rectal carcinoma should be high. In this material there were 20 tumours of the large intestine, 12 being adenocarcinomas of the rectum. In only 6 instances was the sex given (4 males and 2 females) and the ages ranged from 25–59 with a mean of 41 years. In no instance were bilharzia ova noted although they have been seen in two benign rectal papillomata. It should be remembered however that *S. mansoni* is less common in the Gold Coast than *S. haematobium* and heavy infections of *S. mansoni* in contradistinction to *S. haematobium* have not been recorded. In spite of the arguments against schistosomiasis being responsible for bladder carcinoma the present writer considers that it cannot definitely be excluded as an aetiological agent with the information existing at present.

*Adamantinoma*

Twenty-two adamantinomas of the jaw were seen. The sex was given in 14, there being 10 males (ages varying from 12–53 wi th a mean of 32 years) and 4

females (ages ranging from 15–50 with a mean of 35 years). The age incidence agrees with that described by Willis (1953) and no conclusions can be drawn from the sex incidence.

*Carcinoma of the thyroid gland*

Eighty-three specimens of thyroid tissue had been submitted for histological diagnosis. Carcinomatous change was considered to have occurred in 13 instances (11 were papillary adenocarcinoma and 2 specimens were aberrant thyroid tissue removed from the lateral aspect of the neck and reported as malignant.) In addition a solid teratoma and a dermoid cyst were seen. Apart from 2 foetal adenomata the remaining 66 specimens showed the histopathology of the colloid type of adenomatous goitre. Degenerative and cystic changes were common but there was a noticeable absence of lymphocytic infiltration. Neither Riedel's struma nor Hashimoto's disease were seen. The majority of the patients were from the Northern Territories. Toxic symptoms were rarely recorded. The age and sex incidence was available in 46 biopsies and is shown in Table V.

TABLE V.—*Age and Sex Incidence in Thyroid Disease*

| | Simple goitre | | | Carcinoma | | |
|---|---|---|---|---|---|---|
| Sex | Number | Age range | Mean age | Number | Age range | Mean age |
| Females | 33 | 16–50 | 36 | 5 | 30–52 | 43 |
| Males | 7 | 19–50 | 39 | 1 | — | 50 |

It is known that endemic goitre is common in the Navrongo and Bawku areas of the Northern Territories of the Gold Coast but there are no records of its occurrence in the Colony. Annual reports by various medical officers from these districts have emphasised its frequency in the out-patient departments and proposed iodisation of salt as a preventive measure. The aetiology of endemic goitre in South Africa (1955) and Sierra Leone (Wilson, *et al* 1954) has been discussed and is outside the present scope of this paper. It is probable, however, that the incidence of carcinoma of the thyroid will be found to be high in these areas in the Gold Coast and there is a fruitful field for research on this problem. No information is available on the incidence of cretinism or deaf mutism.

*Carcinoma of the pancreas*

Eleven were recorded. In the 10 instances where the sex was noted the tumours were all in males. The ages ranged from 18–60 with a mean of 41 years. In view of the fact that Kwashiorkor is common in the Gold Coast and that it primarily affects the pancreas it was thought that the incidence of carcinoma might be high. This would not appear to be so. Unfortunately there is no information available on the incidents of fibrosis of the pancreas and this condition was considered an aetiological agent in the cause of death in only one subject in the autopsy records, a male aged 30 years. Haemorrhagic pancreatitis has been recorded as a cause of death in 4 instances all in young adult males.

The calcification and fibrosis of the pancreas described by Zuidema (1955) in Indonesian patients and considered to be due to protein deficiency would not appear to be common in the Gold Coast. Fibrocystic disease of the pancreas would also appear to be uncommon. No evidence has been produced to implicate nutritional disease in the aetiology of carcinoma of the pancreas.

*The less common carcinomas*

There was little of note in the nasopharyngeal tumours. There were 10 carcinomas of the testis, 3 were unclassified and the remainder were seminoma (5) interstitial cell tumour (1) and chorionepithelioma (1). In addition there were 2 solid teratomas and one sarcoma in a boy aged 17 years.

In the kidney there were 2 hypernephromas and 2 papillary adenocarcinomas. Wilm's embryoma was the most common type of tumour of the kidney and is considered in its appropriate group.

Cancer of the lung would appear to be rare. In the Gold Coast pipe smoking is generally considered to be more popular than cigarette smoking especially with older men and women, a number of whom are heavy smokers. Cigarette smoking is however, common and is increasing in amount but relatively few of the population would be classed as heavy smokers. Smog of course is not seen but, especially in the North, wood smoke is constantly inhaled and charcoal braziers are employed. Carbon monoxide poisoning may be a cause of unexplained deaths in huts in the cold season. Little more can be said on the lung cancer—smoking controversy but it will be interesting to note if the incidence of lung cancer increases in the future. Two simple lipomas of the pleura were seen in the biopsy material.

Cholecystitis and gallstones are rare in the Gold Coast (Edington, 1956). This is surprising when the high incidence of infectious diseaes and haemolytic anaemias is remembered. Carcinoma of the gall bladder was also rare in the material reviewed as was carcinoma of the lachrymal gland.

### *Group II*

*Tumours of non-haemopoietic mesenchymal tissues*

Considerable difficulty was experienced in classifying the tumours seen in this group. Sarcoma would appear to have been more common in the pre-1948 autopsy and biopsy material and consequently the tissues available for study were limited. In addition the details given in many biopsy specimens were meagre, clinical examination was not possible and follow up results virtually *nil*. In view of these deficiencies it was difficult in a number of instances to classify the tumours as benign or malignant. The tumours causing the most anxiety were the fibromas or fibromyxomas showing considerable cellular activity and in particular, certain of the angiomatous tumours (sclerosing haemangioma, Kaposi's sarcoma, angiosarcoma and the active highly vascularised fibrous tissue seen in certain chronic uhcers). The most common of the simple tumours seen in this group were fibroma (105) uterine fibroids (64), angioma (47), giant cell tumours of bone, tendon sheaths and gum (45). Neurofibromatosis was seen in 20 instances.

In the autopsy material a meningioma caused death in 2 instances and a cavernous angioma of the brain in one. The types of sarcoma seen are shown in Table VI. The high number unclassified has already been explained and the great majority of these from the description available were probably fibrosarcomas.

The sex was given in 32 of the 48 fibrosarcomas. There were 20 males (ages ranging from 4 months to 65 years, mean 27) and 12 females (ages ranging from

TABLE VI.—*Types of Sarcoma Seen in* 4395 *Autopsies and* 1000 *Malignant Biopsy Specimens*

| | | | |
|---|---|---|---|
| Unclassified | 56 | Kaposis sarcoma | 10 |
| Fibrosarcoma | 48 | Chondrosarcoma | 5 |
| Angiosarcoma | 24 | Rhabdomyosarcoma | 5 |
| Osteogenic sarcoma | 16 | Sarcoma botryoides | 5 |
| Total | 169 | | |

7 months to 53 years, mean 35). The most commonly recorded sites were thigh (8), uterus (5), desmoid tumour of the abdominal muscles (4), fibromyxomatous type of tumour in the retroperitoneal tissues (4), groin (4) and buttocks (3). It is of interest to note the occurrence of a sarcoma of the breast (1), heart (1), and stomach (1).

The most common site of the osteogenic sarcomas was in the jaw region and usually occurred in children aged about 7 years.

Osteoclastomas (45) have been excluded from the list of malignant tumours seen as it was found impossible to segregate accurately these tumours from the benign tumours of tendon sheaths and giant cell epulides on histological examination alone in the absence of clinical and radiological information. Osteoclastomas, however, would appear to be more common in the Gold Coast than in England (Eyre-Brook, 1956). These tumours were predominantly found in women in the third decade (sex ratio 6 : 1).

The angiosarcomas and Kaposi's tumours were most commonly sited on the lower limbs. In one autopsy the cause of death was given as Kaposi's sarcomatosis with multiple lesions in the small intestine. As has already been pointed out the histopathological classification of these tumours in the absence of careful clinical studies is difficult and the types of angiomatous tumours seen in the Gold Coast African should be the subject of special investigation in the future.

### *Group III*

### *Tumours of haemopoietic tissues*

The types of malignant disease seen are shown in Table VII.

TABLE VII.—*Tumours of the Haemopoietic Tissues Seen in* 4395 *Autopsies and* 1000 *Biopsy Specimens*

| Type of tumour | Biopsy specimens | Autopsies | Total |
|---|---|---|---|
| Lymphosarcoma and lymphatic leukaemia | 28 | 17 | 45 |
| Hodgkin's disease | 18 | 12 | 30 |
| Reticulosarcoma | 17 | 5 | 22 |
| Unclassified | 11 | 2 | 13 |
| Myelomatosis | 6 | 1 | 7 |
| Leukaemia, myeloid | 2 | 3 | 5 |
| unclassified | — | 5 | 5 |
| monocytic | 2 | — | 2 |
| Total | 84 | 45 | 129 |

In the 13 unclassified tumours were two cases of histiocytic medullary reticulosis, one of Letterer Siwe's disease and one of mycosis fungoides which has already been described (Ashworth and Edington, 1954).

Lymphosarcoma was seen in 37 instances and the sex was noted in 23, there being 19 males (ages ranging from 4–59 with a mean of 20 years) and 4 females (ages ranging from 6–30 with a mean of 12 years). The most common sites were the upper abdominal glands and the maxillary region, young children especially being affected. There were 8 cases of lymphatic leukaemia.

The sex was only noted in Hodgkin's disease in 13 instances there being 8 males and 5 females, the mean ages being 36 and 29 years respectively.

There was little of interest to note in the remaining tumours apart from two reticulosarcomas of the retroperitoneal tissues and one of the small intestine. Reticulosarcomas occurred in an older age group when compared with lymphosarcoma, the mean age in 8 males being 43 years. It is considered that the incidence of leukaemia is much too low as the diagnosis is usually made on haematological findings and consequently these cases would not be considered in this review.

Giant follicular lymphoma, polycythaemia and lipoid storage disease have yet to be diagnosed in the Gold Coast.

*Group IV*

*Tumours of neural tissue*

Malignant tumours of neural tissue with the exception of the retinoblastoma are rarely diagnosed in the Gold Coast. The types of tumour seen are shown in Table VIII.

TABLE VIII.—*Tumours of Neural Tissue*

| | | | |
|---|---|---|---|
| Retinoblastoma | 18 | Chromaffinoma | 1 |
| Glioma | 3 | Cartoid body tumour | 1 |
| Neurileumenoma | 3 | Neuroblastoma | 1 |

The sex in 13 of the retinoblastomas was females 7 (ages $1\frac{1}{2}$–6 with a mean of 3 years, 4 months) and males 6 (ages 2–8 with a mean of $4\frac{1}{2}$ years). One neuroblastoma (mediastinal) was seen in a newly-born male baby and one astrocytoma involving the third ventricle and frontal lobes of the brain was seen at autopsy in a boy aged 13 years.

*Group V*

*Sundry special tumours*

The types of malignant tumours seen are shown in Table IX.

TABLE IX.—*Sundry Malignant Tumours Seen in* 4395 *Autopsy and* 1000 *Biopsy Specimens*

| Type | Autopsy | Biopsy | Total |
|---|---|---|---|
| Malignant melanoma | 3 | 60 | 63 |
| Chorionepthelioma | 1 | 20 | 21 |
| Teratoma | 2 | 6 | 8 |
| Nephroblastoma | 5 | 2 | 7 |
| Unclassified | 2 | — | 2 |
| Total | 13 | 88 | 101 |

Seventy-four specimens of melanoma were seen. Of these only 11 were considered benign and the majority of these were small pigmented tumours of the conjunctivae and were most commonly seen in children. This is understandable as patients with tumours of the eye readily present themselves for treatment and the tumours are noticed when small. Melanotic tumours of the skin were usually described as fungating and it is probable that patients with a small benign melanoma of the skin did not attend for medical treatment. The sex was noted in 33 of the malignant tumours there being 15 males (ages ranging from 45–72 with a mean of 55 years) and 18 females (ages ranging from 36–67 with a mean of 51 years). By far the most common site was the foot especially the plantar aspect and secondary involvement of the inguinal glands was not uncommon in the material. The sites recorded were foot (32), eye (6), abdominal wall (1) and nasal region (1). No sex difference was noted.

Chorionepithelioma was diagnosed in 21 instances. The ages ranged from 19–35 with a mean of 27 years. The majority of these tumours were diagnosed by uterine curettage, in many instances supplemented by a quantitative male toad pregnancy test. Hysterectomy confirmed the diagnosis on four occasions. In two instances the lesions presented in the vaginal wall and in one instance peritoneal secondaries followed a ruptured tubal pregnancy. Hydatidiform mole was not uncommon in the biopsy material, 33 specimens being received. The ages ranged from 19–40 years with a mean of 24. The ratio of hydatidiform mole to chorionepithelioma is much too low and this can probably be explained by the macroscopic diagnosis of a mole being made by medical officers in outstations without recourse to histological examination. Curettage, however, is necessary for the diagnosis of chorionepithelioma and explains its high ratio in the biopsy specimens. It can be stated that the incidence of hydratidiform mole and chorionepithelioma is high in the Gold Coast and the age incidence recorded agrees with that recorded by King (1956) in Chinese.

Eight solid teratoma were seen, the sites recorded being ovary (3), testis (2), thyroid (1) and mediastinum (2). Dermoid cysts were most commonly sited in the ovary (9), orbital region (3) and neck (2). One woman died of peritonitis following a ruptured dermoid cyst of the ovary due to trauma.

Nephroblastoma occurred in the younger age groups. The unclassified tumours in the autopsy material were two embryonic tumours, one situated in the mediastinum of a newly-born child.

### DISCUSSION

The tumours seen in the Gold Coast have been discussed in their relevant groups in the classification adopted. It has been previously noted that the accuracy of the incidence of these tumours is debatable. In an attempt to assess the accuracy of the incidence of the various types of tumours seen the results have been compared with the incidence of malignant disease as seen in Uganda (Davies and Wilson, 1954), in Nigeria (Elmes and Baldwin, 1947) and French West Africa (Camain, 1954). The results are shown in Table X. It is doubtful, in view of the methods of investigation and classification employed by the various writers, if the total figures shown in Table X are 100 per cent comparable. In the Gold Coast, for example, giant cell tumours have been excluded from the total list of malignant tumours whereas in Nigeria they were included. On the other hand leukaemia was not considered in the Nigerian material. Various other discrepancies have been noted but it is not considered that gross inaccuracies have resulted.

TABLE X.—*The Incidence of Various Tumours Compared to the Total Incidence of Malignant Disease Seen in Africans in the Gold Coast, Uganda, Nigeria and French West Africa*

Percentage figures shown in brackets.

| Type of tumours | Gold Coast 1193 | Uganda 734 | Nigeria 1000 | French West Africa 1884 |
|---|---|---|---|---|
| *Carcinoma* | | | | |
| Squamous celled of skin | 166 (13·9) | 100 (13·6) | 122 (12·2) | 235 (12·5) |
| Basal celled of skin | 12 (1·0) | 1 (0·1) | ? | 24 (1·3) |
| Liver | 91 (7·6) | 56 (7·6) | 81 (8·1) | 484 (25·7) |
| Uterus and cervix | 71 (6·0) | 70 (9·5) | 68 (6·8) | 100 (5·3) |
| Breast | 64 (5·4) | 29 (4·0) | 84 (8·4) | 103 (5·4) |
| Stomach | 43 (3·6) | 23 (3·1) | 22 (2·2) | 43 (2·3) |
| Salivary glands | 40 (3·3) | 10 (1·4) | 60 (6·0) | 66 (3·5) |
| Ovary | 33 (2·8) | 36 (4·9) | 27 (2·7) | 36 (2·0) |
| Bladder | 30 (2·5) | 20 (2·7) | 18 (1·8) | 31 (1·6) |
| Prostate | 25 (2·1) | 19 (2·6) | 8 (0·8) | 24 (1·3) |
| Adamantinoma | 22 (1·9) | 10 (1·4) | 18 (1·8) | ??50 (2·7) |
| Large intestine | 20 (1·7) | 19 (2·6) | 14 (1·4) | ?? < 23 |
| Nasopharynx | 14 (1·2) | 18 (2·5) | 19 (1·9) | ? |
| Thyroid | 13 (1·1) | 5 (0·8) | 14 (1·4) | 17 (0·9) |
| Pancreas | 11 (0·9) | 7 (1·0) | 7 (0·7) | 13 (0·7) |
| Testis | 10 (0·8) | 1 (0·1) | 7 (0·7) | 5 (0·3) |
| Kidney | 7 (0·6) | 5 (0·8) | 10 (1·0) | 12 (0·6) |
| Lung | 4 (0·3) | 8 (1·0) | 5 (0·5) | 5 (0·3) |
| Sarcoma | 159 (13·3) | 57 (7·8) | 137 (13·7) | 95 (5·0) |
| Kaposi's | 10 (0·8) | 27 (3·7) | 24 (2·4) | 5 (0·3) |
| Osteoclastoma | 45 (3·6)† | ? | 23 (2·3) | ? |
| Lymphosarcoma and lymphatic leukaemia | 45 (3·8) | 37 (5·0) | 53 (5·3) | 119 (6·3) |
| Hodgkins | 30 (2·5) | 7 (1·0) | — | 35 (1·9) |
| Reticulosarcoma | 22 (1·9) | 6 (0·8) | — | 63 (3·3) |
| Myeloma | 7 (0·6) | 2 (0·3) | — | 12 (0·6) |
| Leukaemia | 9 (0·8) | 11 (1·5) | — | 16 (0·8) |
| Retinoblastoma | 18 (1·5) | 3 (0·4) | 21 (2·1) | 13 (0·7) |
| Glioma | 3 (0·3) | — | 3 (0·3) | 8 (0·4) |
| Melanoma | 63 (5·3) | 13 (1·8) | 62 (6·2) | 49 (2·6) |
| Chorionepithelioma | 21 (1·8) | 3 (0·4) | 9 (0·9) | ? |
| Teratoma | 8 (0·7) | 14 (1·9)* | 3 (0·3) | ? |
| Nephroblastoma | 7 (0·6) | 12 (1·6) | 4 (0·4) | ? |

* Fourteen of kidney

† These giant cell tumours are included only to compare the incidence with that of Elmes and Baldwin (1947).

From Table X it will be seen that there is remarkably close agreement in the incidence of carcinoma in these four territories. The incidence of carcinoma of the liver was however much higher in French West Africa. Carcinoma of the larynx was rare excepting French West Africa (0·8 per cent) as was carcinoma of the oesophagus excepting Uganda (2·3 per cent). Carcinoma of the conjuctiva (31) was also more common in French West Africa but these tumours have included squamous celled carcinomas which were recorded as epitheliomas of the orbital area in the Gold Coast (16) and there were also 21 carcinomas of the orbital region recorded in Uganda.

The incidence of sarcomas in the various territories shows some variation and does not agree as closely as the incidence of carcinoma. The difficulty in histological diagnosis of many of these tumours has however been previously discussed and it is considered that the figures therefore, show surprisingly close agreement. It is also considered that this remark applies to tumours described in the remaining three groups shown in Table X. When the various methods utilised by the investigators are considered Table X would suggest that the overall pattern of malignant disease as described in this paper in the Gold Coast is reasonably accurate. It can thus be concluded that carcinoma is common in the Gold Coast and that the most common types met with are the squamous celled carcinoma of the skin, primary liver celled carcinoma, carcinoma of the uterus and breast and surprisingly, fifth in incidence, carcinoma of the stomach. No evidence has been produced to incriminate schistosomiasis as an aetiological agent in the production of carcinoma of the bladder.

Further investigations are required to determine accurately the types and incidence of sarcomas seen in the Gold Coast. Tumours of the haemopoietic tissues are common and should prove a fruitful field of research in the future. Malignant melanoma is also common, and as already stated, trauma would appear to be a precipitating factor in its aetiology. Protective footwear should decrease its incidence.

The figures given in most publications from Africa which consider malignant disease are small when compared to those recorded in Europe but gradually the pattern of malignant disease is emerging. In the four territories considered it shows reasonably close agreement. At present few conclusions can be drawn but it is doubtful if malnutrition plays a great part in the aetiology of malignant disease in tropical Africa. Trauma, chronic ulceration and reticuloendothelial hyperplasia due to multiple infestations and infections may well be the most important predisposing factors.

### SUMMARY AND CONCLUSIONS

Autopsy protocols and biopsy records have been scrutinised in the Medical Research Institute, Accra, and all available material has been examined. The type of malignant disease seen has been recorded in age and sex groups. The results have been compared with the incidence of malignant disease in other parts of tropical Africa and it is concluded that the findings recorded in this paper portray reasonably accurately the incidence of the various malignant tumours which occur in the Gold Coast.

I am most grateful to Professor C. V. Harrison for his advice willingly given on many occasions during the last five years, to Messrs Laryea and Tamakloe for technical assistance and to Dr. E. Akwei, Chief Medical Officer, Gold Coast, for permission to publish.

### REFERENCES

Ashworth, F. and Edington, G. M.—(1954) *W. Afr. med. J.*, (New Series). **3**, 150.
Berman, C.—(1951) 'Primary Carcinoma of the Liver', London (H. K. Lewis & Co.).
Camain, R.—(1954) *Bull. Soc. Path. exot.*, **47**, 614.
Davies, J. N. P. and Wilson, B. A.—(1954) *E. Afr. med. J.*, **31**, 396.

EDINGTON, G. M.—(1956) *Trans. R. Soc. trop. Med. Hyg.* (in press).
ELMES, B. G. T. AND BALDWIN, R. B. T.—(1947) *Ann. trop. Med. Parasit.*, **41**, 321.
EYRE-BROOK, A. L.—(1956) *Proc. R. Soc. Med.*, **49**, 409.
FINDLAY, G. M.—(1950) *J. R. micr. Soc.*, **70**, 166.
GAMBLE, M. E.—(1954) *W. Afr. med. J.* (New Series), 3, 48.
GELFAND, M.—(1950) 'Schistosomiasis in South Central Africa.' Cape Town (Juta & Co.).
KING, G.—(1956) *Proc. R. Soc. Med.* **49**, 381.
MACFIE, J. W. S.—(1922) *Trans. R. Soc. trop. Med. Hyg.* **16**, 156 and 257.
ROSE, J. R.—(1955) *Lancet* i, 107.
South Africa Endemic Goitre in the Union (1955) Department of Nutrition.
WILLIS, R. A.—(1953) 'Pathology of Tumours' London (Butterworth and Co.).
WILSON, D. C., GRUNDY, H. M., STEEL, R. W. AND EDDEY, T. P.—(1954) *Trans. R. Soc. trop. Med. Hyg.*, **48**, 481.
ZUIDEMA, P. J.—(1955) *Docum. med. geog. trop.*, **7**, 229.